# Quality of life and wellbeing among breast cancer patients in Lahore, Pakistan

**DOI:** 10.3389/fonc.2023.1105411

**Published:** 2023-06-29

**Authors:** Fiza Ayub, Tahir Mehmood Khan, Mirza Rafi Baig, Muhammad Usman Amin, Humera Tahir

**Affiliations:** ^1^ Institute of Pharmaceutical Science, University of Veterinary and Animal Science UVAS, Lahore, Pakistan; ^2^ Department of Clinical Pharmacy and Pharmacotherapeutics, Dubai Pharmacy College for Girls, Dubai, United Arab Emirates; ^3^ Department of Pharmacy, Abasyn University, Peshawar, Pakistan; ^4^ Ruth Pfau College of Nutrition Sciences, Lahore Medical and Dental College, Lahore, Pakistan

**Keywords:** quality of Life, breast cancer, Pakistan, symptom prevalence, FACIT-B

## Abstract

**Background:**

Breast cancer has a high incidence rate, emphasizing the necessity of enhanced information on health-related quality of life (HrQOL) in this population of patients. The aim of this study was to identify the factors influencing the QOL experienced by patients in Pakistan.

**Methods:**

A cross-sectional study was conducted on women with breast cancer, and four instruments were used on a random sample of 130 Pakistani women: FACIT-B Version 4 questionnaire,WHO causality assessment scale, Naranjo’s algorithm, and a demographic/clinical characteristics section. Data analysis included descriptive analysis, independent sample t-test, and analysis of variance (ANOVA) test.

**Results:**

The patients’ mean age was 49.10 (standard deviation (SD) 10.89); 98.5% were married. The mean score was 18.34 for physical wellbeing (SD 5.92; interquartile range (IQR) 11), 16.33 for social/family wellbeing (SD 6.3; IQR 11.25), 13.6 for emotional wellbeing (SD 3.55; IQR 6), 17.13 for functional wellbeing (SD 3.73; IQR 6), and 24.86 for breast cancer subscale (SD 3.64; IQR 4). The study found that the age, entitlement, recurrence, marital status, salary, number of doses, duration of cancer treatment, and chemotherapy sessions were significantly related to QOL terms in the assessment of the FACIT-B scale. The WHO causality evaluation scale determined that 78.1% of the responses were “probable” and 20.1% were “possible”. According to Naranjo’s algorithm assessment scale, 80% of adverse drug reactions (ADRs) were “probable”, whereas 18.4% were declared “possible”. Chemotherapy-induced anemia was the most often reported ADR in 64.6% of patients, followed by chemotherapy-induced nausea and vomiting (61.5%).

**Conclusion:**

Healthcare practitioners must acknowledge and take into account the significance of QOL in addition to therapy for breast cancer patients in order to enhance their health. The findings of this study will aid in filling gaps in current unknown knowledge and identifying sites where patients require additional assistance. Because cancer and chemotherapy clearly have a negative impact on individuals’ QOL, oncologists must concentrate on strategies that help cancer patients during their sickness and treatment while also enhancing self-care and QOL. Those with cancer will benefit from emotional wellbeing and adaptation to their disease.

## 1 Introduction

Breast cancer (BC) is the most frequently diagnosed cancer in both developed and developing nations. An estimated 2.3 million new cases were diagnosed in 2020. Worldwide, breast cancer poses a serious threat to the health of women (1). Every year, 1.1 million women are diagnosed with breast cancer, and 410,000 women die from the disease worldwide. It is the fifth leading cause of death worldwide, accounting for 685,000 deaths in 2020 (1).

Women are seen to have an important part in the household. When a woman is confirmed to have breast cancer, her household is affected in some way, whether directly or indirectly. As a result, restoring and improving the quality of life of breast cancer women would have a significant impact on both individual and societal wellbeing (2).

While it is anticipated that early identification, therapy, and advancements in treatment would increase survival rates, issues with the treatment itself might have a severe impact on the quality of life in terms of health. Quality of life (QOL) of patients is now regarded as a crucial factor in the care of breast cancer patients (3, 4).

The QOL is an evaluation of the effects of the disease’s diagnosis, development, and treatment on the social and personal lives of breast cancer patients as well as the effectiveness of their rehabilitation. Quality of life is a multifaceted concept that incorporates physical wellbeing, psychological health, social and cognitive functioning, illness effect, and therapy based on the patient’s life experiences. Health is more than just the absence or presence of sickness; it also involves social and physical functioning (5). Developing nations such as Pakistan appear to have become so intent on the diagnosis and screening difficulties of those individuals who have been diagnosed and those who require active treatment, and cancer survivors appear to be given a lower priority. The prevalence of breast cancer is quickly growing in Pakistan, and as a result, the patient’s quality of life suffers.

Health-related quality of life is associated with an individual’s wellbeing and ability to do everyday tasks that are more likely to be influenced by sickness or a health condition. It is a multifaceted idea that includes physical, social, psychological, and functional wellbeing primarily influenced by cancer diagnosis and treatment (5). Fatigue, soreness, shortness of breath, and bleeding are all symptoms of physical wellbeing. Loss of fertility, sadness, and anxiety are all aspects of psychological health. Social wellbeing comprises participation in and success at activities or others. Functional wellbeing relates to the patients’ functional state and their capacity to perform routine everyday tasks on their own.

Cancer therapies have an impact on a patient’s body image, quality of life, and cognitive skills. Unwanted side effects that impact appearance, such as apparent scars, hair loss, skin discoloration, muscular weakness, loss of or abnormalities in the breasts, and weight fluctuation, cause the survivors to experience negative emotions like anxiety and sadness (6). Not unexpectedly, these significant physical and physiological changes can have a significant impact on a woman’s body image (BI) (7). Body image is defined as a multidimensional construct that includes thoughts and feelings about one’s physical appearance, attractiveness, and competency, as well as one’s perceived state of general health, completeness, functioning, and sexuality (8). BI is a highly subjective experience that occurs as a result of a dynamic interaction between this unique manifestation of being and the social world. Dissatisfaction with one’s “new” physique has a negative impact on several psychological areas for many BC survivors. Following treatment, BI disturbance has been linked to mental distress, anxiety, poor physical health, sexual dysfunction, and a lower quality of life (9).

Some studies have found that several psychological interventions focusing on self-compassion are beneficial to breast cancer survivors (10) (11). Self-compassion is described as being sensitive to one’s own and others’ suffering and making a commitment to attempt to relieve and avoid it. It is connected to mindfulness and is distinguished by six characteristics: sensitivity, compassion, empathy, motivation, discomfort and tolerance. Self-compassion therapies can reduce breast cancer survivors’ anxiety and sadness while also improving their quality of life (7, 12).

According to a qualitative study by Kim et al. (13), the change in one’s appearance is a traumatic and unpleasant event that affects patients’ everyday lives, social events, relationships, and quality of life (7).

Emotional distress is also an important issue in chronic diseases like cancer; the American Psychiatric Association recognized cancer diagnosis as a traumatic stressor capable of causing impairment in various areas of functioning (ability to work and social relationships) due to negative cognitions and mood (14). Patients often respond to the disease’s development and treatment in a variety of ways, and they may experience feelings of sadness and stress (15, 16).

Previous research has revealed that women with breast cancer may have reduced emotional functioning following cancer therapy (17). Given the importance of emotions in the quality of life and health management of breast cancer survivors, it is critical to evaluate individuals’ capacities to understand and control emotions so that they do not become an impediment to health and wellbeing. Psychological research has discovered many capacities, sometimes distinct and sometimes overlapping conceptions, that contribute to efficient emotion control. The terms “emotional intelligence”, “functional coping”, “emotional management”, and “mood repair” all come from the theoretical underpinnings of psychological research and refer to the capacities to understand, control, and effectively utilize emotions to promote health and wellbeing (18).

While early identification and therapy, as well as advancements in treatment, are predicted to improve survival rates, treatment-related issues can have a detrimental influence on health-related quality of life (HrQOL). Today, patient QOL is a major consideration in the cure of breast cancer survivors (19).

Pain, tiredness, anxiety, and depression symptoms are typical difficulties associated with a breast cancer diagnosis and influence 20% to 30% of women with breast cancer prognosis (20). Anger, sadness, depression, low self-esteem, and a lack of emotional support are all associated with psychological distress in breast cancer patients.

For women who have been diagnosed with breast cancer, social support is a key indicator for coping with challenging situations and adjusting to the psychological and social demands imposed on them (21). According to research, women with breast cancer report that their ability to cope and adjust is favorably connected with how close their spouses and family members are to them (22–24). HRQOL is favorably correlated with the availability of social support, such as the existence of supportive family, friends, and social networks (23).

The health-related quality of life statistics is intended to provide information about patient experiences with treatment, to inform clinical risk management regarding the best course of action, and perhaps to forecast diagnosis. Such connections, however, were only found in studies undertaken in Western nations (25, 26). However, it is currently unknown in developing countries like Pakistan whether healthcare decision is impacted by health-related QOL assessments or whether this impact varies depending on the stage of cancer or the kind of treatment (27, 28).

Many prior studies have partially addressed the multiple potentially important aspects when determining the health-related quality of life and have been confined to subsets of breast cancer women, such as early-stage breast cancer, or have omitted the oldest women. As a result, we conducted research using the Functional Assessment of Cancer Therapy-Breast (FACIT-B) questionnaires to identify the factors influencing the QOL experienced by patients in Pakistan.

## 2 Materials and methods

### 2.1 Study design and population

A questionnaire-based quantitative and cross-sectional study on breast cancer patients It was conducted in January 2021 at the INMOL Hospital Lahore.

The study population comprised 130 patients who would undergo follow-up care for breast cancer at the hospital. The sample size was calculated by clincalc.com (29) (sample size calculator). The confidence interval was 95%, and the margin of error was 5%. The eligibility criteria of our study were patients >18 years of age, patients with breast cancer who are able to perform any activities or are not on bed rest, and those who agreed to participate in the study.

### 2.2 Data collection

To identify the factors affecting the health-related quality of life, the data were collected using the FACIT-B Version 4 questionnaire composed of 37 cancer-specific items asking respondents to rate how true each statement is for the last 7 days (30). It was divided into five primary QOL domains: Physical Wellbeing (PWB; energy, pain, nausea, and physical discomfort), Social/Family Wellbeing (SWB; social support, interpersonal relationship, marriage, sexual function, and family), Emotional Wellbeing (EWB; psychological distress, negative feelings, and positive feelings), Functional Wellbeing (FWB; work, sleep, and daily living capability), and breast cancer subscale (BCS; shortness of breath, body pain, hair loss, depression, and weight loss). The BCS contains items specific to the interest of women with breast cancer. Patient responses were converted to scores on a scale of 0 (not at all) to 4 (very much) for each domain. The FACIT has been shown to have high internal consistency reliability and has been well-validated (31). Cronbach’s alpha (kr-20) was used for the Urdu version of FACIT (32). The following were also used: Pearson’s raw score-to-measure correlation = 1.00 and Cronbach alpha (kr-20) Pearson’s raw score reliability = 0.90.

WHO causality assessment scale (33) and Naranjo’s algorithm (34) were used for the assessment of reported symptoms such as hair loss, pain, nausea/vomiting, weight loss, numbness/muscle weakness, diarrhea, constipation, loss of appetite, shortness of breath, fever/chills, and sleep disturbances. Naranjo’s algorithm consists of 10 objective questions with three response options: yes, no, and don’t know. The drug’s causality can be graded as “definite”, “probable”, “possible”, or “unlikely” based on the scores. The WHO causality assessment scale is categorized into “certain”, “probable”, “possible”, “unlikely”, “conditional/unclassified”, and “assessable/unclassifiable”.

### 2.3 Statistical analysis

The entire questionnaire was assigned a serial number to ensure traceability. Coding of the responses was performed, and the data collected were processed using Statistical Package for Social Science (SPSS) software program for Windows version 20.0.

The data were analyzed using the appropriate parametric statistics such as frequencies, mean, median, standard deviation, interquartile range (IQR), and cross-tabulation. Apart from this, the association of independent variables like age, weight, height, entitlement, stage of cancer, recurrence, number of children, any changes in dosage form or regimen, medication therapy, and surgery were explored using parametric statistics such as chi-square, independent t-test, and analysis of variance (ANOVA) test. The statistical significance level was 0.05 with a confidence interval of 95%. The chi-square test was used to compare categorical variables between groups; ANOVA and independent t-test were used to compare continuous variables across groups, with pairwise mean differences evaluated using ANOVA contrasts.

### 2.4 Ethical consideration

This study protocol approval of the institutional review board of the University of the Veterinary and Animal Sciences Lahore (Ref No. 147/IRC/BMR) was obtained first followed by the approval of the INMOL hospital administration. The study’s purpose was explained to the patients clearly before information collecting from them. All survey respondents were also asked for their verbal agreement. All data were kept private in accordance with ethical standards.

## 3 Results

**Table 1** displays the clinical and demographic characteristics of the study sample (n = 130). The patients’ mean age was 49.10 (standard deviation (SD) 10.89), mean weight was 66.80 (SD 14.49), and mean height was 155.61 (SD 7.09). The majority of the patients were married, 98.5% (n = 128). Of the patients, 6.9% (n = 9) had stage 1 cancer, 24.6% (n = 32) were at stage 2, 46.2% (n = 60) were at stage 3, and 22.3% (n = 29) were at stage 4. Approximately 69.2% (n = 90) were receiving chemotherapy, 10.8% (n = 14) were receiving chemotherapy and hormonal agents, 16.2% were receiving radiation and chemotherapy, and 3.8% (n = 5) were receiving hormonal agents, radiation, and chemotherapy treatment. None of them were receiving only hormonal agents. Among the total of 130 patients, 89.2% (n = 116) underwent surgery, and 10.8% (n = 14) did not undergo surgery.

**Table 1:** Clinical and demographic data of respondents.

Characteristics	N	%
**Age**		
25–34	10	7.7
35–44	34	26.2
45–54	45	34.6
55–64	30	23.1
65+	11	8.5
		
**Weight**		
38–47	9	6.9
48–57	31	23.8
58–67	20	15.4
68–77	40	30.8
78–87	21	16.2
88+	9	6.9
		
**Height**		
140–149	22	16.9
150–159	68	52.3
160–169	35	26.9
170+	5	3.8
		
**Marital status**		
Single	2	1.5
Married	128	98.5
**Stage of cancer**		
1	9	6.9
2	32	24.6
3	60	46.2
4	29	22.3
**Number of children**		
0	9	6.9
1	8	6.2
2	29	22.3
3	28	21.5
4	36	27.7
5	10	7.7
6+	10	7.7
**Recurrence**		
No	124	95.4
Yes	6	4.6
**Family entitled**		
Entitled	14	10.8
Semi-entitled	85	65.4
Not entitled	31	23.8
**Salary**		
Lower income	14	10.8
Lower middle income	84	64.6
Upper middle income	32	24.6
**Number of doses**		
0–5	23	17.7
6–10	54	41.5
11–15	30	23.1
16–20	16	12.3
21–25	7	5.4
**Any changes in dosage form or regimen?**		
No	57	43.8
Yes	73	56.2
**Duration (years)**		
1–4	118	90.8
5–8	9	6.9
9+	3	2.3
**Medication therapy**		
Chemotherapy	90	69.2
Chemotherapy+hormonal agents	14	10.8
Radiation+chemotherapy	21	16.2
Radiation+chemotherapy+hormonal agents	5	3.8
Hormonal agents	0	0
**Surgery**		
Yes	116	89.2
No	14	10.8

### 3.1 Comparing QOL in relation to demographic characteristics of breast cancer patients

By ANOVA and independent t-test, we found that patients of different age groups showed significant differences in physical wellbeing, functional wellbeing, and social wellbeing. In the emotional wellbeing and breast cancer subscale, we found no significant difference between age groups. Patients with entitlement had a significant difference in terms of PWB. Similarly, patients receiving a higher number of doses of medicines had higher scores in terms of PWB. However, patients who received any medication therapy (chemotherapy, hormonal, radiation, and combination of radiation, chemotherapy, and hormonal) and surgery and had any stage of cancer (1–4) had no effect on their quality of life. The details are presented in **Table 2**.

**Table 2:** Comparing QOL in relation to demographic characteristics of breast cancer patients.

Variables	PWB	EWB	FWB	SWB	BCS
**Age**					
25–34	12 (3.3)	11.8 (2.8)	21.1 (2.3)	20.3 (4.4)	26.6 (3.9)
35–44	18.1 (6.2)	13.7 (3.5)	17.9 (3.2)	17.8 (5.6)	24.9 (2.9)
45–54	18.2 (5.5)	13.3 (3.6)	16.3 (3.2)	14.4 (7.0)	24.7 (4.1)
55–64	20.1 (5.8)	14.0 (3.3)	17.2 (4.2)	16.3 (5.9)	24.2 (3.8)
65+	20.2 (4.9)	14.6 (4.5)	13.7 (2.9)	15.3 (5.5)	25.1 (2.9)
p-Value	0.03	0.3	0.00	0.03	0.5
Weight					
38–47	17.0 (5.8)	12.5 (2.5)	15.5 (4.3)	14.1 (6.6)	23.6 (4.9)
48–57	18.3 (6.5)	13.5 (3.7)	18.0 (2.9)	17.3 (5.9)	25.3 (3.1)
58–67	17.7 (6.6)	12.5 (3.8)	17.1 (4.5)	18.0 (4.9)	24.6 (3.6)
68–77	19.7 (5.9)	13.9 (3.8)	16.9 (3.9)	15.7 (6.7)	24.8 (4.0)
78–87	16.9 (4.2)	13.8 (3.1)	17.0 (3.8)	16.0 (7.3)	24.5 (2.8)
88+	18.3 (5.8)	15.7 (2.0)	16.4 (2.4)	14.8 (5.6)	26.2 (3.8)
p-Value	0.56	0.26	0.54	0.51	0.7
Height					
140–149	17.1 (3.9)	13.5 (3.2)	18.4 (3.5)	15.4 (6.1)	25.0 (4.0)
150–159	18.5 (6.4)	13.4 (3.6)	17.0 (3.5)	15.8 (6.4)	24.4 (3.9)
160–169	18.7 (6.0)	14.1 (3.6)	16.1 (4.0)	17.4 (6.1)	25.4 (2.7)
170+	18.0 (5.6)	12.0 (3.2)	19.6 (2.3)	19.4 (4.5)	25.4 (3.8)
p-Value	0.76	0.77	0.06	0.38	0.6
Entitlement					
Entitled	22.4 (5.5)	14.6 (4.3)	17.0 (3.5)	17.1 (6.6)	25.0 (2.0)
Semi-entitled	17.7 (5.9)	13.6 (3.5)	17.0 (3.5)	15.5 (6.2)	24.9 (3.5)
Not-entitled	18.4 (5.7)	13.2 (3.2)	17.2 (4.2)	17.9 (6.2)	24.5 (4.2)
p-Value	0.04	0.57	0.97	0.16	0.84
Stage of cancer					
1	17.5 (5.5)	12.5 (2.8)	18.4 (4.1)	18.6 (6.7)	24.2 (3.0)
2	17.2 (5.6)	13.5 (3.5)	17.6 (3.0)	16.9 (6.7)	24.1 (4.6)
3	19.1 (6.0)	13.7 (3.7)	16.5 (4.0)	15.4 (6.2)	25.3 (3.5)
4	18.2 (6.2)	13.9 (3.4)	17.3 (3.5)	16.7 (5.7)	24.9 (2.7)
p-Value	0.49	0.76	0.38	0.42	0.52
Recurrence					
Yes	23.1 (6.1)	14.6 (4.4)	15.6 (4.8)	16.8 (5.6)	24.8 (3.4)
No	18.1 (5.8)	13.5 (3.5)	17.2 (3.6)	16.3 (6.3)	24.8 (3.6)
p-Value	0.04	0.47	0.32	0.84	0.98
Marital status					
Single	11.0 (0.0)	11.0 (1.4)	23.0 (1.4)	20.0 (0.0)	22.5 (4.9)
Married	18.4 (5.8)	13.6 (3.5)	17.0 (3.6)	16.2 (6.3)	24.9 (3.6)
p-Value	0.00	0.29	0.02	0.35	0.00
No. of children					
0	17.2 (5.7)	13.4 (3.0)	19.8 (3.2)	16.5 (7.7)	23.7 (4.4)
1	16.0 (7.0)	13.6 (3.3)	17.1 (3.6)	15.2 (7.9)	24.3 (5.0)
2	17.2 (5.6)	13.4 (3.6)	18.3 (3.9)	17.6 (6.3)	25.6 (4.0)
3	19.3 (6.1)	13.8 (3.5)	16.7 (3.3)	16.8 (5.8)	24.6 (2.3)
4	18.7 (5.6)	13.0 (4.0)	16.3 (3.5)	16.1 (5.7)	24.2 (3.4)
5	21.5 (5.9)	13.6 (3.5)	17.4 (4.1)	16.5 (5.8)	25.5 (2.6)
6+	16.9 (5.7)	15.8 (2.0)	14.6 (2.9)	12.2 (6.7)	26.1 (4.8)
p-Value	0.33	0.58	0.02	0.42	0.55
Any changes in dosage form or regimen?					
Yes	19.5 (6.1)	13.1 (3.4)	16.8 (3.9)	16.4 (6.0)	24.6 (3.4)
No	16.8 (5.3)	14.2 (3.6)	17.5 (3.4)	16.1 (6.6)	25.1 (3.9)
p-Value	0.01	0.1	0.32	0.8	0.51
Medication therapy					
Chemotherapy	17.6 (5.6)	13.7 (3.4)	17.5 (4.0)	16.4 (6.1)	24.9 (3.6)
Chemotherapy+hormonal agents	21.5 (6.7)	14.4 (4.1)	16.0 (3.4)	18.6 (6.1)	26.0 (3.5)
Radiation+chemotherapy	19.3 (6.6)	12.6 (3.7)	16.3 (2.3)	14.0 (6.5)	24.1 (3.7)
Radiation+chemotherapy+hormonal agents	16.8 (2.4)	12.8 (2.1)	16.8 (3.4)	17.0 (7.3)	24.0 (3.8)
Hormonal agents	0	0	0	0	0
p-Value	0.09	0.44	0.38	0.18	0.45
Surgery					
No	20.3 (4.4)	16.6 (2.7)	17.5 (2.9)	14.0 (7.5)	24.7 (2.7)
Yes	18.1 (6.0)	13.2 (3.4)	17.0 (3.8)	16.6 (6.1)	24.8 (3.7)
p-Value	0.18	0.15	0.64	0.15	0.86
Salary					
Lower income	21.2 (5.7)	13.7 (3.9)	16.9 (3.4)	17.8 (5.7)	25.2 (2.2)
Lower middle income	17.8 (5.9)	13.7 (3.6)	17.0 (3.6)	15.2 (6.2)	25.0 (3.6)
Upper middle income	18.2 (5.7)	13.2 (3.2)	17.5 (4.0)	18.4 (6.0)	24.2 (4.1)
p-Value	0.13	0.8	0.76	0.03	0.5
Number of Doses					
0–5	15.4 (5.2)	13.1 (3.5)	17.0 (3.5)	15.3 (6.9)	24.8 (3.5)
6–10	17.9 (5.9)	13.8 (3.8)	17.7 (4.1)	16.5 (6.0)	25.0 (3.7)
11–15	18.7 (5.9)	13.0 (3.4)	16.4 (3.4)	17.0 (6.2)	24.8 (3.9)
16–20	20.1 (5.1)	14.9 (2.5)	16.3 (2.2)	13.5 (5.4)	24.4 (3.5)
21–25	25.2 (2.9)	13.2 (3.5)	17.7 (4.4)	21.2 (5.6)	24.2 (3.4)
p-value	0.01	0.46	0.5	0.06	0.96
Duration (years)					
1–4	24.8 (3.6)	13.5 (3.5)	17.2 (3.7)	16.0 (6.3)	24.8 (3.6)
5–8	24.2 (3.9)	13.2 (3.0)	16.6 (3.8)	19.5 (5.3)	24.2 (3.9)
9+	26.6 (1.1)	16.6 (4.0)	14.0 (3.4)	19.6 (4.5)	27.3 (1.5)
p-Value	0.02	0.31	0.3	0.17	0.44

QOL, quality of life; PWB, physical wellbeing; EWB, emotional wellbeing; FWB, functional wellbeing; SWB, social/family wellbeing; BCS, breast cancer subscale.

### 3.2 Average score of different domains of QOL among breast cancer patients

The mean score was 18.34 for physical wellbeing (SD 5.92; IQR 11), 16.33 for social/family wellbeing (SD 6.3; IQR 11.25), 13.6 for emotional wellbeing (SD 3.55; IQR 6), 17.13 for functional wellbeing (SD 3.73; IQR 6), and 24.86 for breast cancer subscale (SD 3.64; IQR 4). The details are mentioned in **Table 3**.

**Table 3:** Average score of different domains of QOL among breast cancer patients.

	Mean	Median	IQR	Minimum value	Maximum value	Std. deviation
**Physical wellbeing**	18.34	19	11	5	28	5.92
**Social/family wellbeing**	16.33	17.5	11.25	3	24	6.3
**Emotional wellbeing**	13.6	14	6	5	23	3.55
**Functional wellbeing**	17.13	17	6	9	27	3.73
**Breast cancer subscale**	24.86	24	4	14	35	3.64

QOL, quality of life; IQR, interquartile range.

### 3.3 Causality assessment of individual adverse drug reaction

**Table 4** shows the causality assessment of individual adverse drug reactions (ADRs) using both the WHO causality assessment scale and Naranjo’s causality assessment scale. The WHO causality evaluation scale determined that 78.1% of the responses were “probable” and 20.1% were “possible”. As re-challenge was not undertaken in any of the patients, there were no “certain” ADRs. According to Naranjo’s algorithm assessment scale, 80% of ADRs were “probable” whereas 18.4% were declared “possible”. Chemotherapy-induced anemia was the most often reported ADR in 64.6% of patients, followed by chemotherapy-induced nausea and vomiting (61.5%).

**Table 4:** Causality assessment of individual adverse drug reaction.

Adverse drug reaction	Number of adverse drug reactions					
	WHO causality assessment scale			Naranjo’s algorithm		
	Possible	Probable	Total	Possible	Probable	Total
Hair loss	7	63	70	4	66	70
Pain	0	130	130	0	130	130
Diarrhea	28	1	29	28	1	29
Constipation	12	0	12	12	0	12
Nausea/vomiting	13	67	80	13	67	80
Loss of appetite	8	52	60	8	52	60
Anemia	20	64	84	13	71	84
Weight loss	15	114	129	15	114	129
Shortness of breath	30	17	47	27	20	47
Fatigue	20	40	60	20	40	60
Total	153	548	701	140	561	701

### 3.4 Distribution of patients with breast cancer undergoing chemotherapy in terms of quality of life based on the number of chemotherapy sessions

**Table 5** shows the connection between QOL and the frequency of chemotherapy (CT) cycles. As indicated, the majority of patients had average QOL. There was a significant association between QOL and the frequency of CT sessions (df = 4, *X* ^2 =^ 11.89, *p* < 0.05).

**Table 5:** Distribution of patients with breast cancer undergoing chemotherapy in terms of quality of life based on the number of chemotherapy sessions.

Quality of life number of chemotherapy sessions	Poor	Average	Good	Total	p-Value
<2 sessions	21	29	20	70	0.01
3–5 sessions	12	11	5	28	
>6 sessions	10	5	17	32	
**Total**	43	45	42	130	

### 3.5 Percentage of patients receiving respective drugs

**Figure 1:**
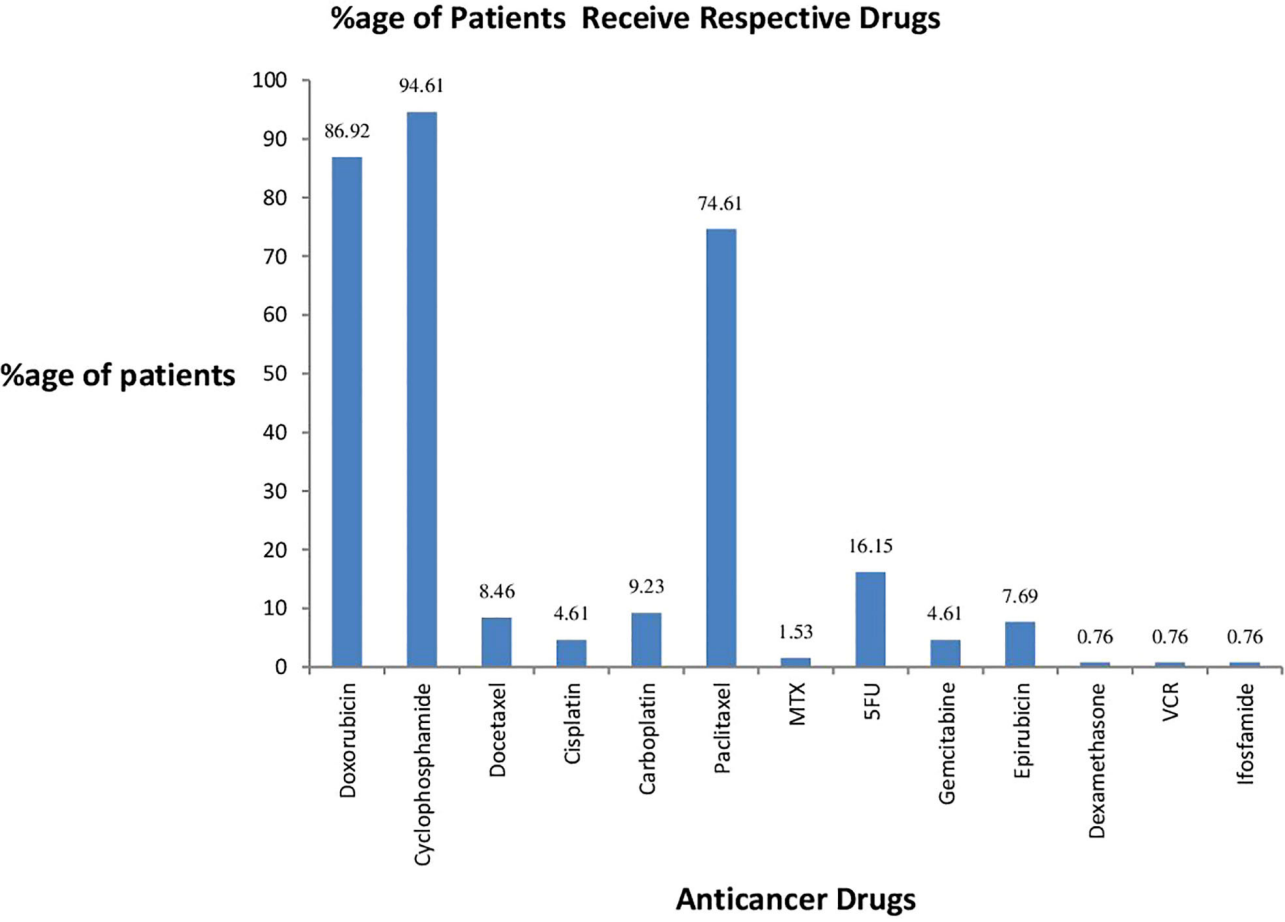
Percentage of patients receiving respective drugs.

**Figure 1** shows the percentage of patients receiving respective drugs during their treatment. Cyclophosphamide was received by most of the patients at 94.61%. Doxorubicin was the second-highest drug received by the patients at 86.92%. Of the patients, 74.61% received paclitaxel drugs. Dexamethasone, vincristine (VCR), and ifosfamide were received by a small number of patients, 0.76%.

## 4 Discussion

Quality of life is a subjective notion that is evaluated from the patient’s point of view. Oncological treatment is based on the use of effective therapy approaches while maintaining a good quality of life. For a better understanding, the present study looked at the quality of life of breast cancer patients as well as the factors that influence their QOL. According to this study, the quality of life was shown to be related to age, weight, height, marital status, entitlement, salary/income level, stage of cancer, medication therapy, surgery, duration of cancer, cost of the treatment, and chemotherapy sessions. Only age, entitlement, recurrence, marital status, salary, number of doses, and duration of cancer were significantly related to QOL terms in the assessment of the FACIT-B scale. We found that patients lack emotional support in all age groups and at all stages of breast cancer, which worsens the patient’s quality of life. These results offer a scientific foundation for creating a comprehensive program that includes these elements, particularly social and emotional support, to enhance the QOL of breast cancer survivors in Pakistan and worldwide.

In the current study, we found that physical wellbeing, functional wellbeing, and social wellbeing were significantly affected by the age variable, which is similar to the findings of Park et al. (35). On the contrary, previous studies (Huang et al., 2017) and (Daldoul et al., 2018) examined the influence of socio-demographic characteristics on the quality of life in women with breast cancer and found that QOL was not age-dependent. However, some studies also suggested that older patients had better QOL than younger ones. According to a US study, younger breast cancer patients have more difficulty with partner relationships, sexual function, and body image, as well as less effective coping mechanisms. They are also more likely to undergo chemotherapy than older patients. As a result, they had a poorer overall quality of life than older breast cancer patients (36) (37). These studies’ discrepancies may be explained by variations in sample size, population background, subject source, and change.

Similar to previous studies, we discovered that marital status was significantly related to functional wellbeing among breast cancer patients (38). Moreover, no significant relationship between the Emotional and Social/Family Wellbeing dimension and marital status was found in this study, and this condition is influenced by the fact that Pakistani women have lower expectations for their sexual lives and are more reserved when responding to questions in this module because of their cultural and social practices. The QOL of breast cancer survivors is greatly improved by a stable family dynamic and regular interactions with friends and neighbors, two particular indicators of social support. These results suggest that emotional support from friends and family, as well as other social relationships, is essential for surviving breast cancer and recovering from it. It was also discovered that women who did not have children were significantly worse psychologically than women who have children. According to the findings given by Ramadas et al. (2015), the quality of life of breast cancer patients was greater in those who were married and lived with their families.

Many studies have shown that income has an impact on the quality of life and all of its aspects (39–41). We found the quality of life only in terms of social wellbeing was significantly related to income. As most of the patients belong to the low-middle income group, government subsidies and other supporting methods may improve the overall quality of life of the patients and help in their recovery (42).

Consistent with the finding of a previous study (43), no significant association is found between quality of life and stage of cancer, surgery, chemotherapy, hormonal therapy, radiation, and a combination of all of them. The stage of the disease is a significant factor in treatment planning as well as providing care and support. In more advanced stages, patients have increased discomfort, physiological issues, and decreased physical activity as a result of chemotherapy, which ultimately has an impact on the quality of life. Therefore, cancer survivors who have had oncological treatment must obtain a long-term rehabilitation plan as well as psychiatric counseling in order to lessen their suffering or restore their health. Another notable discovery was the existence of a strong link between the QOL of breast cancer patients and the frequency of chemotherapy cycles. As a result, the frequency of chemotherapy treatments enhanced the mean QOL score in patients, and they had a good quality of life. In support of this conclusion, Shabanlooie demonstrated in a research titled “Analyzing the QOL of Participants Receiving Chemotherapy Directed to Specified Tabriz Hospitals” that there existed a direct association between QOL and frequency of chemotherapy cycles (44). Furthermore, the findings of previous studies were consistent with the findings of the current investigation (45, 46). However, Aghabarari et al. discovered an indirect (negative) association between patient QOL and the frequency of chemotherapy treatments (47). Kornblith’s study found no significant variation in cancer patients’ QOL before, during, and after chemotherapy (48).

The introduction of additional antineoplastic medicines has extended the arsenal of oncological treatment during the last few decades, but it has also increased the prevalence of ADRs. When medications are used in greater or different communities than those evaluated in early clinical trials, new ADRs are frequently reported. This usually happens within 3 years of the medicine hitting the market. As a result, reporting and documenting ADRs become critical in defining a drug’s side effect profile. This may assist to prevent such accidents in the future. An ethical medical profession necessitates precise and fair medication knowledge. This is only achievable with a rigorous medication safety monitoring program (49). A well-functioning hospital-based reporting scheme may be useful in revealing knowledge about possible drug-use issues in a facility. Problems can be recognized and remedied as a consequence of these efforts, leading to ongoing improvement in patient care (50).

The majority of patients (69.2%) only had chemotherapy, while some required additional therapies such as the combination of chemotherapy and hormonal therapy (10.8%), radiation and chemotherapy (16.2%), or a combination of chemotherapy, hormonal therapy, and radiotherapy (3.8%). The various treatment strategies chosen are determined according to a number of criteria, including the stage of the disease, the cost of the treatment plan, and patient and physician-related considerations. This research took into account ADRs that occurred solely as a result of chemotherapy.

Except for hair loss and anemia, which were assessed as “possible” with a lower level of causality by the WHO scale but were judged as “probable” with a higher level of causality by Naranjo’s algorithm, shortness of breath was assessed as “possible” with a higher level of causality by the WHO scale but was judged as “probable” with a lower level of causality by Naranjo’s algorithm. The majority of the reactions in this study showed a similar causality assessment by both the WHO causality assessment scale and Naranjo’s algorithm. There were no “certain” reactions since no patients were re-challenged. Because of a variety of co-administered medicines, the causality grade remained low. There were no “unlikely” reactions since the investigator was educated in pharmacovigilance protocols, and such complaints were avoided.

Using Naranjo’s algorithm to analyze the causality of ADRs, we discovered that 80% of cases showed probable links and 18.4% indicated possible links, although Khandelwal and colleagues reported 100% and Goyal and colleagues reported 61% of probable scores using the same scale (51, 52).

The most prevalent ADRs in our patients were nausea and vomiting. These were also reported to be the most prevalent ADRs in previous investigations (53, 54). Chemoreceptor trigger zone (CTZ) activation is the most prevalent mechanism of chemotherapy-induced nausea and vomiting (55, 56). Because vomiting is a prevalent side effect of cancer chemotherapy, techniques for preventing and managing vomiting in cancer chemotherapy patients should be developed.

Hair loss is typically connected with drugs such as doxorubicin, daunorubicin, docetaxel, and cyclophosphamide (Hinds and Thomas, 2008). In our study, a total of 53.84% of individuals had alopecia, which is almost similar to 51% and 58% reported in earlier studies (57–59).

Despite advances in precision medicine that have improved cancer prognosis, treatment costs have skyrocketed (60, 61). Furthermore, the loss of assets following a diagnosis is not confined to treatment expenditures, and it has a long-term impact on patient wellbeing income (62). As a result, doctors must be aware of their patients’ financial stress and provide them with appropriate treatments (63).

Healthcare practitioners must acknowledge and take into account the significance of QOL in addition to therapy for breast cancer patients in order to enhance their health. The findings of this study will aid in filling gaps in current unknown knowledge and identifying sites where patients require additional assistance. Because cancer and chemotherapy clearly have a negative impact on individuals’ QOL, oncologists must concentrate on strategies that help cancer patients during their sickness and treatment while also enhancing self-care and QOL. Those with cancer will benefit from emotional wellbeing and adaptation to their disease. Planned education programs that address patients’ needs, provide verbal encouragement to patients, and incorporate recommendations for pain management in patient care are all important strategies for developing QOL among breast cancer patients.

### 4.1 Limitations and recommendations

This is perhaps among the very few recent studies aiming to explore the HrQOL of breast cancer patients in Pakistan and the factors associated with them.

Due to practical constraints, in order to assess the patients’ long-term HrQOL, we did not keep in touch with them.

Unfortunately, our study is not an interventional study, and we could not perform any intervention according to the present condition of the patients because breast cancer patients are critical and suffer from physiological trauma. Future studies like (64–66) on physiological and physical interventions should be designed to develop the best methods for improving patients’ quality of life. The sample size was small, and it is possible that statistical power was not enough to find the other factors that affect QOL. Future studies are required to find more factors that affect the QOL of cancer patients.

Due to scheduling constraints, data were obtained only from only one cancer hospital, which is the main center of Punjab, Pakistan. However, further information from other cancer hospitals in different regions of Pakistan is required.

One significant drawback of the study is that we only examined 10 adverse drug reactions, leaving out many more ADRs that may have occurred in the patients who received chemotherapy sessions during their treatment. At the specific timeframe, only these ADRs were reported. However, future studies should explore more ADRs so that appropriate pharmacovigilance data can be generated.

## 5 Conclusion

In conclusion, this study provided baseline information on the QOL and wellbeing of patients. The study found that the age, entitlement, recurrence, marital status, salary, number of doses, duration of cancer treatment, and chemotherapy sessions were significantly related to QOL terms in the assessment of the FACIT-B scale. The finding of this study also reveals that emotional support is required in all age groups and at all stages of breast cancer patients to improve their quality of life. Chemotherapy-induced anemia was the most often reported ADR in 64.6% of patients, followed by chemotherapy-induced nausea and vomiting (61.5%). Cancer and chemotherapy clearly have a negative impact on patients’ quality of life, so oncologists must consider different interventions that improve self-care and QOL and support cancer patients around their illness and chemotherapy. The government of Pakistan should also take note of the recommended interventions present in the study implications. Data reporting should also be improved so that in the future we will recommend better interventions for the patients.

## Data availability statement

The raw data supporting the conclusions of this article will be made available by the authors, without undue reservation.

## Ethics statement

The studies involving human participants were reviewed and approved by Institutional review board of the University of the Veterinary and Animal Sciences Lahore. The patients/participants provided their written informed consent to participate in this study. Written informed consent was obtained from the individual(s) for the publication of any potentially identifiable images or data included in this article.

## Author contributions

FA participated in the design of the study, performed data collection and analysis, and drafted the manuscript. TK, MB, MA, and HT participated in the design of the study and revised and helped to draft the manuscript. All authors contributed to the article and approved the submitted version.

## References

[1] WHO. World fact sheets cancers. In: Globocan 2020, (WHO) vol. 419. (2020). p. 1–2. Available at: https://gco.iarc.fr/today/data/factsheets/populations/900-world-fact-sheets.pdf.

[2] Nayak MG George A Shashidhara YN Nayak BS. Symptom interference and relation between the domains of quality of life among cancer patients of tertiary care hospital. Indian J Palliat Care (2019) 25(4):575–9. doi: 10.4103/IJPC.IJPC_139_19 PMC6812431 31673215

[3] Potter S Thomson HJ Greenwood RJ Hopwood P Winters ZE. Health-related quality of life assessment after breast reconstruction. Br J Surg (2009) 96(6):613–20. doi: 10.1002/bjs.6605 19434704

[4] Salonen P Tarkka M-T Kellokumpu-Lehtinen P-L Åstedt-Kurki P Luukkaala T Kaunonen M. Telephone intervention and quality of life in patients with breast cancer. Cancer Nurs. (2009) 32(3):177–90. doi: 10.1097/NCC.0b013e31819b5b65 19295417

[5] Fallowfield L. What is quality of life. What is. (2009) 2.

[6] Triberti S Savioni L Sebri V Pravettoni G. eHealth for improving quality of life in breast cancer patients: a systematic review. Cancer Treat Rev (2019) 74:1–14. doi: 10.1016/j.ctrv.2019.01.003 30658289

[7] Sebri V Durosini I Mazzoni D Pravettoni G. The body after cancer: a qualitative study on breast cancer survivors&rsquo; body representation. Int J Environ Res Public Health (2022) 19. doi: 10.3390/ijerph191912515 PMC9566341 36231811

[8] Zhao W Chong YY Chien WT. Effectiveness of cognitive-based interventions for improving body image of patients having breast cancer: a systematic review and meta-analysis. Asia-Pacific J Oncol Nurs (2023) 10(4):100213. doi: 10.1016/j.apjon.2023.100213 PMC10120298 37089782

[9] Sebri V Durosini I Triberti S Pravettoni G. The efficacy of psychological intervention on body image in breast cancer patients and survivors: a systematic-review and meta-analysis. Front Psychol (2021) 12:611954. doi: 10.3389/fpsyg.2021.611954 33732184 PMC7957010

[10] Kirby JN. Compassion interventions: the programmes, the evidence, and implications for research and practice. Psychol Psychother Theory Res Pract (2017) 90(3):432–55. doi: 10.1111/papt.12104 27664071

[11] Neff KD Germer CK. A pilot study and randomized controlled trial of the mindful self-compassion program. J Clin Psychol (2013) 69(1):28–44. doi: 10.1002/jclp.21923 23070875

[12] Boquiren VM Esplen MJ Wong J Toner B Warner E. Exploring the influence of gender-role socialization and objectified body consciousness on body image disturbance in breast cancer survivors. Psycho-Oncology (2013) 22(10):2177–85. doi: 10.1002/pon.3271 23512273

[13] Kim I-R Cho J-H Choi E-K Kwon I-G Sung Y-H Lee J-E. Perception, attitudes, preparedness and experience of chemotherapy-induced alopecia among breast cancer patients: a qualitative study. Asian Pacific J Cancer Prev (2012) 13(4):1383–8. doi: 10.7314/APJCP.2012.13.4.1383 22799336

[14] Arnaboldi P Lucchiari C Santoro L Sangalli C Luini A Pravettoni G. PTSD symptoms as a consequence of breast cancer diagnosis: clinical implications. Springerplus. (2014) 3:1–7. doi: 10.1186/2193-1801-3-392 25105089 PMC4124104

[15] Bahrami M Mohamadirizi S Mohamadirizi S. Hardiness and optimism in women with breast cancer. Iran J Nurs Midwifery Res (2018) 23(2):105. 29628957 10.4103/ijnmr.IJNMR_200_16 PMC5881226

[16] Nakatani Y Iwamitsu Y Kuranami M Okazaki S Yamamoto K Watanabe M. Predictors of psychological distress in breast cancer patients after surgery. Kitasato Med J (2013) 43:49–56.

[17] Pourfallahi M Gholami M Tarrahi MJ Toulabi T Kordestani Moghadam P. The effect of informational-emotional support program on illness perceptions and emotional coping of cancer patients undergoing chemotherapy. Support Care Cancer (2020) 28:485–95. doi: 10.1007/s00520-019-04842-w 31065837

[18] Durosini I Triberti S Savioni L Sebri V Pravettoni G. The role of emotion-related abilities in the quality of life of breast cancer survivors: a systematic review. Int J Environ Res Public Health (2022) 19. doi: 10.3390/ijerph191912704 PMC9566755 36232004

[19] Mokhatri-Hesari P Montazeri A. Health-related quality of life in breast cancer patients: review of reviews from 2008 to 2018. Health Qual Life Outcomes (2020) 18(1):1–25. doi: 10.1186/s12955-020-01591-x 33046106 PMC7552560

[20] Hamood R Hamood H Merhasin I Keinan-Boker L. Chronic pain and other symptoms among breast cancer survivors: prevalence, predictors, and effects on quality of life. Breast Cancer Res Treat (2018) 167(1):157–69. doi: 10.1007/s10549-017-4485-0 28861642

[21] Sebri V Mazzoni D Triberti S Pravettoni G. The impact of unsupportive social support on the injured self in breast cancer patients. Front Psychol (2021) 12:722211. doi: 10.3389/fpsyg.2021.722211 34616337 PMC8488137

[22] Ahn S Kim S Zhang H. Changes in depressive symptoms among older adults with multiple chronic conditions: role of positive and negative social support. Int J Environ Res Public Health (2017) 14(1):16. 10.3390/ijerph14010016 PMC5295267 28035968

[23] Michael YL Berkman LF Colditz GA Holmes MD Kawachi I. Social networks and health-related quality of life in breast cancer survivors: a prospective study. J Psychosom Res (2002) 52(5):285–93. doi: 10.1016/S0022-3999(01)00270-7 12023125

[24] Breuer N Sender A Daneck L Mentschke L Leuteritz K Friedrich M. How do young adults with cancer perceive social support? a qualitative study. J Psychosoc Oncol (2017) 35(3):292–308. doi: 10.1080/07347332.2017.1289290 28145814

[25] Von Ah DM Russell KM Carpenter J Monahan PO Zhao Q Tallman E. Health-related quality of life of African American breast cancer survivors compared to healthy African American women. Cancer Nurs (2012) 35(5):337. 22228394 10.1097/NCC.0b013e3182393de3 PMC3326198

[26] Lei Y-Y Ho SC Cheng A Kwok C Lee C-KI Cheung KL. Adherence to the world cancer research Fund/American institute for cancer research guideline is associated with better health-related quality of life among Chinese patients with breast cancer. J Natl Compr Cancer Netw (2018) 16(3):275–85. doi: 10.6004/jnccn.2017.7202 29523666

[27] Bottomley A Pe M Sloan J Basch E Bonnetain F Calvert M. Analysing data from patient-reported outcome and quality of life endpoints for cancer clinical trials: a start in setting international standards. Lancet Oncol (2016) 17(11):e510–4. doi: 10.1016/S1470-2045(16)30510-1 27769798

[28] Blazeby JM Avery K Sprangers M Pikhart H Fayers P Donovan J. Health-related quality of life measurement in randomized clinical trials in surgical oncology. J Clin Oncol (2006) 24(19):3178–86. doi: 10.1200/JCO.2005.05.2951 16809741

[29] Clinc Calc.com. Sample size calculator. Available at: https://clincalc.com/stats/samplesize.aspx.

[30] Webster K Cella D Yost K. The functional assessment of chronic illness therapy (FACIT) measurement system: properties, applications, and interpretation. Health Qual Life Outcomes [Internet]. (2003) 1:79. doi: 10.1186/1477-7525-1-79 14678568 PMC317391

[31] Censoni P. Certified translation - summary, Vol. 59. (2018). p. 42908.

[32] Yang Y Green SB. Coefficient alpha: a reliability coefficient for the 21st century? J Psychoeduc Assess (2011) 29(4):377–92. doi: 10.1177/0734282911406668

[33] Organization WH Oreagba IA Usman SO Olayemi SO Oshikoya KA Opanuga O. The use of the WHO-UMC system for standardized case causality assessment. Uppsala Uppsala Monit Cent (2014) 48(3):194–203.

[34] Naranjo CA Busto U Sellers EM Sandor P Ruiz I Roberts EA. A method for estimating the probability of adverse drug reactions. Clin Pharmacol Ther (1981) 30(2):239–45. doi: 10.1038/clpt.1981.154 7249508

[35] Park B-W Lee S Lee AR Lee K-H Hwang SY. Quality of life differences between younger and older breast cancer patients. jbc (2011) 14(2):112–8. doi: 10.4048/jbc.2011.14.2.112 PMC3148538 21847405

[36] Kwan ML Ergas IJ Somkin CP Quesenberry CP Neugut AI Hershman DL. Quality of life among women recently diagnosed with invasive breast cancer: the pathways study. Breast Cancer Res Treat (2010) 123:507–24. doi: 10.1007/s10549-010-0764-8 PMC2935682 20140494

[37] Hamer J McDonald R Zhang L Verma S Leahey A Ecclestone C. Quality of life (QOL) and symptom burden (SB) in patients with breast cancer. Support Care Cancer (2017) 25(2):409–19. doi: 10.1007/s00520-016-3417-6 27696078

[38] Konieczny M Cipora E Sygit K Fal A. Quality of life of women with breast cancer and socio-demographic factors. Asian Pac J Cancer Prev (2020) 21(1):185–93. doi: 10.31557/APJCP.2020.21.1.185 PMC7294011 31983183

[39] Pandey M Thomas BC SreeRekha P Ramdas K Ratheesan K Parameswaran S. Quality of life determinants in women with breast cancer undergoing treatment with curative intent. World J Surg Oncol (2005) 3(1):1–7. doi: 10.1186/1477-7819-3-63 16188030 PMC1261539

[40] Benedict C Fisher S Schapira L Chao S Sackeyfio S Sullivan T. Greater financial toxicity relates to greater distress and worse quality of life among breast and gynecologic cancer survivors. Psycho-Oncology (2022) 31(1):9–20. doi: 10.1002/pon.5763 34224603 PMC9809212

[41] Huang H-Y Tsai W-C Chou W-Y Hung Y-C Liu L-C Huang K-F. Quality of life of breast and cervical cancer survivors. BMC Womens Health (2017) 17(1):30. doi: 10.1186/s12905-017-0387-x 28403855 PMC5389170

[42] Ayub F Sulaiman AK Rabbani I Lee KS Goh KW Nadeem MF. Price variation among registered brands of anti-cancer medicines available in Pakistan. Malaysian J Pharm (2022) 8(1):42–56. doi: 10.52494/EXVW6975

[43] Daldoul A Khechine W Bhiri H Ammar N Bouriga R Krir MW. Factors predictive of quality of life among breast cancer patients. Asian Pac J Cancer Prev (2018) 19(6):1671–5. 10.22034/APJCP.2018.19.6.1671 PMC6103599 29938464

[44] Shabanlooie R Motaarefi H. (2005). Quality of life among chemotherapy patients admitted to selected hospitals in tabriz, in: Proceedings of the 1st Congress of Quality of life in Tarbiat Modares University, . p. 53.

[45] Damodar G Smitha T Gopinath S Vijayakumar S Rao YA. Assessment of quality of life in breast cancer patients at a tertiary care hospital. Arch Pharm Pr. (2013) 4(1):15–20. doi: 10.4103/2045-080X.111577

[46] Hürny C Bernhard J Castiglione-Gertsch M Coates AS Peterson HF Gelber RD. Impact of adjuvant therapy on quality of life in women with node-positive operable breast cancer. Lancet (1996) 347(9011):1279–84. doi: 10.1016/S0140-6736(96)90936-8 8622502

[47] Aghabarari M Ahmadi F Mohammadi I Hajizadeh E Varvani Fa. AV F. Physical, emotional and social dimension of quality of life among breast cancer women under chemotherapy. Iran J Nurs Res (2006) 1(3):55–65.

[48] Kornblith AB Lan L Archer L Partridge A Kimmick G Hudis C. Quality of life of older patients with early-stage breast cancer receiving adjuvant chemotherapy: a companion study to cancer and leukemia group b 49907. J Clin Oncol (2011) 29(8):1022. doi: 10.1200/JCO.2010.29.9859 21300923 PMC3068052

[49] Organization WH International drug monitoring: the role of the hospital, report of a WHO meeting [held in Geneva from 18 to 23 November 1968]. (Geneva: WHO) (1969).

[50] Baniasadi S Habibi M Haghgoo R Karimi Gamishan M Dabaghzadeh F Farasatinasab M. Increasing the number of adverse drug reactions reporting: the role of clinical pharmacy residents. Iran J Pharm Res IJPR. (2014) 13(1):291–7. PMC3985255 24734083

[51] Khandelwal S Bairy KL Vidyasagar MS Chogtu B Sharan K. Adverse drug reaction profile of cancer patients on chemotherapy in a tertiary care hospital. Int J Pharma Bio Sci (2015) 6(2):233–44.

[52] Wahlang JB Laishram PD Brahma DK Sarkar C Lahon J Nongkynrih BS. Adverse drug reactions due to cancer chemotherapy in a tertiary care teaching hospital. Ther Adv Drug Saf. (2017) 8(2):61–6. doi: 10.1177/2042098616672572 PMC5315222 28255433

[53] Jamali J Dayo A Memon N Mughal U Khatri MA Muhammad S. Assessment of patient‘s response about adverse drug reactions receiving AC (Adriamycin, cyclophosphamide) Therapy : a survey research. J Pharm Res Int (2021).

[54] Koliyakodu PS Sudhakar R Kurian MM Jishnu RK Kumar R Lalitha V. A prospective observational study on evaluation of chemotherapy induced adverse drug reactions in cancer patients in a tertiary care hospital. Indian J Pharm Pract (2021) 14(1). doi: 10.5530/ijopp.14.1.2

[55] Lau PM Stewart K Dooley M. The ten most common adverse drug reactions (ADRs) in oncology patients: do they matter to you? Support Care Cancer (2004) 12(9):626–33. 10.1007/s00520-004-0622-5 15064936

[56] Stewart DJ. Nausea and vomiting in cancer patients. In: Nausea and vomiting: recent research and clinical advances. CRC Press (2017). p. 178–204.

[57] Surendiran A Balamurugan N Gunaseelan K Akhtar S Reddy KS Adithan C. Adverse drug reaction profile of cisplatin-based chemotherapy regimen in a tertiary care hospital in India: an evaluative study. Indian J Pharmacol (2010) 42(1):40. doi: 10.4103/0253-7613.62412 20606836 PMC2885639

[58] Poddar S Sultana R Sultana R Akbor MM Azad MAK Hasnat A. Pattern of adverse drug reactions due to cancer chemotherapy in tertiary care teaching hospital in Bangladesh. Dhaka Univ J Pharm Sci (2009) 8(1):11–6.

[59] Saraswat N Chopra A Sood A Kamboj P Kumar S. A descriptive study to analyze chemotherapy-induced hair loss and its psychosocial impact in adults: our experience from a tertiary care hospital. Indian Dermatol Online J (2019) 10(4):426–30. 10.4103/idoj.IDOJ_471_18 PMC6615375 31334063

[60] Miller KD Nogueira L Mariotto AB Rowland JH Yabroff KR Alfano CM. Cancer treatment and survivorship statistics, 2019. CA Cancer J Clin (2019) 69(5):363–85. doi: 10.3322/caac.21565 31184787

[61] Schoen C Doty MM Robertson RH Collins SR. Affordable care act reforms could reduce the number of underinsured US adults by 70 percent. Health Aff. (2011) 30(9):1762–71. doi: 10.1377/hlthaff.2011.0335 21900668

[62] Carrera PM Kantarjian HM Blinder VS. The financial burden and distress of patients with cancer: understanding and stepping-up action on the financial toxicity of cancer treatment. CA Cancer J Clin (2018) 68(2):153–65. doi: 10.3322/caac.21443 PMC6652174 29338071

[63] Currow D Aranda S. Financial toxicity in clinical care today: a “menu without prices.” Med J Aust (2016) 204(11):397. doi: 10.5694/mja16.00182 27318391

[64] Durosini I Triberti S Sebri V Giudice AV Guiddi P Pravettoni G. Psychological benefits of a sport-based program for female cancer survivors: the role of social connections. Front Psychol (2021) 5516. doi: 10.3389/fpsyg.2021.751077 PMC8664561 34899491

[65] Savioni L Triberti S Durosini I Sebri V Pravettoni G. Cancer patients’ participation and commitment to psychological interventions: a scoping review. Psychol Health (2022) 37(8):1022–55. doi: 10.1080/08870446.2021.1916494 33966548

[66] Sebri V Durosini I Mazzoni D Pravettoni G. Breast cancer survivors’ motivation to participate in a tailored physical and psychological intervention: a qualitative thematic analysis. Behav Sci (Basel). (2022) 12(8):271. 36004842 10.3390/bs12080271 PMC9404874

